# Evaluation of the Effects of Photobiomodulation on Bone Density After Placing Dental Implants: A Pilot Study Using Cone Beam CT Analysis

**DOI:** 10.3390/clinpract15030064

**Published:** 2025-03-17

**Authors:** Ruxandra-Elena Luca, Alessandro Del Vecchio, Ioana-Roxana Munteanu, Mădălin-Marius Margan, Carmen Darinca Todea

**Affiliations:** 1 Department of Oral Rehabilitation and Dental Emergencies, Faculty of Dentistry, “Victor Babes” University of Medicine and Pharmacy, Eftimie Murgu Square No. 2, 300041 Timisoara, Romania; luca.ruxandra@umft.ro (R.-E.L.); todea.darinca@umft.ro (C.D.T.); 2 Interdisciplinary Research Center for Dental Medical Research, Lasers and Innovative Technologies, Revolutiei 1989 Avenue No. 9, 300070 Timisoara, Romania; 3 Department of Oral and Maxillofacial Sciences, Sapienza University of Rome (Italy), 00185 Rome, Italy; alessandro.delvecchio@uniroma1.it; 4 Department of Functional Sciences, Discipline of Public Health, Victor Babes University of Medicine and Pharmacy, 300041 Timișoara, Romania; margan.madalin@umft.ro; 5 Center for Translational Research and Systems Medicine, Faculty of Medicine, Victor Babes University of Medicine and Pharmacy, 300041 Timișoara, Romania

**Keywords:** photobiomodulation, cone beam CT 2, bone mineral density, implant

## Abstract

**Background:** One of the parameters of maximum interest regarding the quality of the intraoral hard tissues is represented by the bone density, with direct clinical implications. The evaluation of this extremely important clinical parameter can be achieved by several imaging methods, of which the most known in dentistry is represented by the cone beam computed tomography (CBCT). **Objectives**: The purpose of the study is to obtain a quantitative analysis of bone mineral density changes in patients who underwent treatments of photobiomodulation (PBM), as complementary to a surgical approach in oral surgery and implantology. **Methods:** The study included the retrospective analysis of maxillary cone beam computed tomography of 28 patients without pathology or medication known to affect bone metabolism or its qualitative and quantitative properties. All patients from the study group followed the same laser PBM treatment protocol after placing dental implants; the PBM protocol implied the intraoral use of a gallium aluminum arsenide laser (GaAlAs) of 808 nm, 450 mW, in pulsed mode, administering an energy of 6 J in 3 points corresponding to each inserted dental implant—mesial, distal, and apical—totaling 18 J/implant. Treatment sessions were performed immediately postoperatively and at a subsequent distance of 48 h for 2 weeks (a total of eight sessions). For every patient, bone density was analyzed before and after PBM treatment, in the same areas of interest, within the same anatomical landmarks. A comparison was also made between the results obtained for the anterior maxilla and the posterior maxilla. All the measurements made were analyzed statistically, the results being presented in the dedicated section. **Results:** Based on the data analysis, the comparison between the lasered and non-lasered groups reveals that patients who underwent PBM showed a statistically significant improvement in bone mineral density, with the mean increasing from 530.91 HU before treatment to 842.55 HU after treatment (*t*-test: *p* < 0.001). In contrast, the non-lasered group showed no significant improvement, with a slight decrease in bone mineral density, as the mean dropped from 495.19 HU before treatment to 462.16 HU after treatment (*t*-test: *p* = 0.47). **Conclusions:** The study demonstrated results with statistical significance regarding the mineral bone density improvement of patients who underwent laser PBM treatment. This positive effect of laser therapy has been shown, both at the level of the vestibular cortical bone and at level of the trabecular bone, independent of the patient’s sex, for the anterior maxilla and at the lateral areas also.

## 1. Introduction

The field of guided bone regeneration and implant-supported therapies have acquired a massive spread in dentistry in recent years due to the growing need to restore lost tissue, at a level and quality that allow for complex and complete oral rehabilitation. Our previous research in this direction has focused on the possibilities of adding the benefits of laser therapy to current surgical techniques. This method of treatment, also known as photobiomodulation (PBM), is very extensive and still difficult to evaluate clinically. Nevertheless, there are some very well-known effects of PMB, such as anti-inflammatory effects, accelerating the regeneration process by providing cell proliferation and differentiation, being simultaneously a non-invasive and painless therapeutic strategy [1,2,3,4]. PBM consists of using light sources (coherent lasers or non-coherent LEDs) with a wavelength between 405 and 1100 nm and an energy density of less than 10 J/cm^2^ in the direct application of coherent lasers, which can penetrate the tissues in a non-destructive mode, with negligible thermal sound or vibration effects, and induce photochemical reactions resulting in the modulation of different cellular processes [4,5].

Numerous studies use different parameters in order to obtain experimental or clinical effects. The molecular mechanisms by which PBM induces different biological responses have not been fully clarified. Therefore, identifying the most appropriate and effective wavelengths, beam type, and parameters (energy density, irradiation mode, exposure time) able to positively impact the cell behavior represents a priority to obtain consistent results allowing the design of effective treatment protocols [4,6,7,8,9,10,11,12].

Not only does the problem of standardizing PBM treatments cause difficulties but also another challenge, namely, quantifying the effects of this type of treatment in an objective, precise, and reproducible way. Regarding the jaw bones, a qualitative and quantitative clinical evaluation method is based on computed tomography (CT) or reducing disadvantages, such as image artifacts, high cost, the complexity of the examination, and high dose radiation, on cone beam computed tomography (CBCT), which was introduced in dentistry since 1998 [13,14]. CBCT provides a sub-millimetric accuracy assessment of bone quantity, with good image sharpness and minimal distortion, using lower radiation doses and costs than medical computed tomography [15]. Among the many advantages and applications that have increased its popularity, the opportunity to measure the bone mineral density (BMD) is also counted. BMD is the most important objective parameter in the diagnosis and follow-up of systemic bone diseases, such as osteoporosis, being able to provide information on the quality of bone tissue, giving doctors the opportunity to review treatment options. Bone fragility and strength are direct proportional to the amount and organization of mineral substance, calcium, and bone phosphate. Bone density, therefore, has an impact on the success of dental implants, a reduced bone density increasing the risk of failure. In addition, the poor quality and quantity of tissue bone are reported as risk factors associated with excessive resorption and affecting the healing process after implant placement [16,17].

Multi-slice computed tomography (MSCT), which is the modality of choice for measurements bone density, has limitations in measuring the number of trabeculae (Tb.N), the average thickness of trabeculae (Tb.Th), and the distance between trabeculae (Tb.Sp) due to the resolution (150–300 μm in the plane and 300–500 μm between planes) being larger than the trabecular dimensions (50–200 μm) [18]. The CBCT digital volume, on the other hand, has a cylindrical shape and is composed of 265 voxels. The isotropic voxels of small size with a wide range of gray values improve image quality and ensure the accuracy of dimensional measurements, as well as the subsequent visualization of the anatomical structures [19]. The cylindrical digital volume obtained by rotating a beam of cone-shaped X-rays around the patient’s head is called the field of view (FOV). The FOV size remains the most important scanning factor regarding radiation dose limitation and image quality improvement [20]. Unlike CT, the voxels of CBCT images do not represent absolute values of gray (Hounsfield unit or HU) [21]. Various research has been carried out to assess the reliability of this imaging tool in measurements of bone density. Large amounts of scattered X-rays and artifacts are cited as the main causes for lower reliability CBCT in the assessment of bone mineral density [22]. CBCT scanners are operated at a lower kVp and mA than the MSCT, resulting in a low signal-to-noise ratio [23]. Higher noise level will cause many more discrepancies and a larger standard deviation of gray values voxel [24,25]. In addition, since the volume beam in CBCT is proportionally larger than the collimated one in MSCT, the effect of these artifacts is greater [23,26]. However, other studies have shown a correlation high between CT and CBCT values in terms of grayscale; though, therefore, it is suggested that voxel values of CBCT can be used, in some limits, to estimate bone mineral density [27,28,29,30,31].

Knowing all these data and limitations from the literature, our scientific approach aims to address together the following two extremely important concepts: on the one hand, how PBM can influence the quality of the jaw bones, and on the other hand, if the CBCT is capable of providing us with an objective quantification, by measuring bone density, of the effects of laser therapy. The purpose of the study is, therefore, to obtain a quantitative analysis of bone mineral density in patients who underwent treatments of photobiomodulation. We aim to analyze not only the effects of the PBM treatment at the level of the maxillary bones but also the applicability and accuracy of CBCT in the assessment of bone mineral density.

## 2. Materials and Methods

The study included retrospective analysis of maxillary cone beam computed tomography, which was conducted with the consent of the ethical committee of the “Victor Babes” University of Medicine and Pharmacy Timisoara, Romania, no. 93/21 January 2020. Patient’s CBCTs selected from the database made available in this regard were subjected to a selection process based on the following criteria.

Inclusion criteria:

- Relatively healthy patient, who does not have associated general pathologies known to influence bone metabolism or drug treatments with the same effect;
- Existence of an initial CBCT investigation, prior to a surgical intervention;
- Patient underwent an oral surgery treatment (implant insertion), followed by a PBM treatment protocol of same dosage as shown below;
- The existence of a control CBCT investigation, after 8–12 months;
- The initial and the control CBCT were performed using the same device with the same acquisition parameters.

Exclusion criteria:

- Patients having different general conditions associated with changes in bone metabolism (diabetes mellitus, osteoporosis, bisphosphonate treatment, malignancy, renal or liver malfunctions, endocrine disorders);
- Smokers;
- Patients that did not respect the time indicated for carrying out the control CBCT
- the two CBCT volumes, initial and control, have different sizes or different acquisition parameters;
- Patients receiving bone regeneration treatments in the studied area.

**Figure 1:**
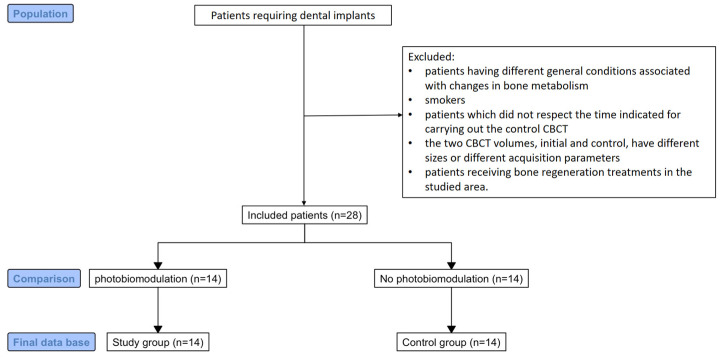
A flowchart showing patient selection and study groups.

The study aimed to analyze CBCTs of comparable or identical FOV to diminish the error of measurements. We note that all these investigations were performed before the present study, under precise medical indications, and without scientific implications. Patients signed the informed consent regarding the surgical procedures and also the PBM treatment, this being the routine protocol in such cases in the dental office. The application of the selection criteria caused a severe reduction in the number of included patients, from over 40 to 14 patients cone beam computed tomography, 8 women and 6 men, aged between 24 and 74 years, which represented the study group. The inclusion of a control group in this study arose from the need to observe variations in the HU index measured on CBCT in the absence of surgical interventions at the level of the maxillary bones. In this regard, a database of another doctor in the research team, who does not perform laser PBM therapy, was used. Therefore, the control group included 14 patients who met the same inclusion criteria, minus the laser PBM treatment (Figure 1).

**Figure 2:**
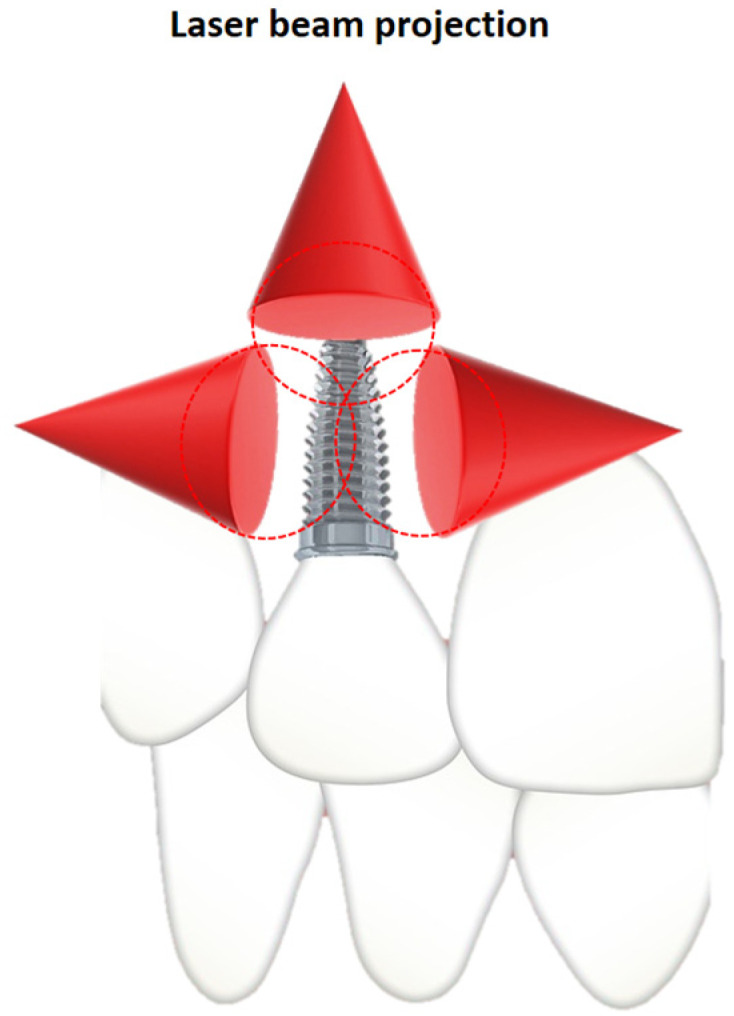
Scheme representing the 3 points of application of laser radiation—mesial, distal, and apical—next to each inserted dental implant.

All patients from the study group followed the same laser PBM treatment protocol after the completion of the intervention: the PBM protocol implied the intraoral use of a gallium aluminum arsenide laser (GaAlAs) (MID-laser; Serial no 8110131-4) of 808 nm, 450 mW, in pulsed mode, administering an energy of 6 J in 3 points corresponding to each inserted dental implant—mesial, distal, and apical—totaling 18 J/implant (Figure 2). Treatment sessions were performed immediately postoperatively and at a subsequent distance of 48 h, for 2 weeks (a total of 8 sessions), observing all the precautions of laser safety (both the patient and the medical team wore safety glasses protection corresponding to the used wavelength). The laser safety officer and the maintenance team are responsible for periodically checking the equipment, including the correspondence between the set laser parameters and the energy dose released by the laser. The laser irradiation protocol that was implemented in these patients was previously confirmed by animal studies of our research group, which is the reason for choosing this therapeutic dose. Moreover, specialized studies in the literature use similar doses for this purpose.

To minimize factors that could influence the measurements and for consistency, we analyzed the data recorded with a single type of equipment (ProMax 3D CBCT (Planmeca, Helsinki, Finland)), using same image acquisition parameters (voltage, current intensity, voxel size, and field of view): 89 kV voltage, intensity a current of 4–8 mA, cylindrical field of view (FOV) of 80 mm both in diameter as well as in height, voxel size of 120 μm. All images obtained were recorded using the digital imaging and communications format in medicine (DICOM). In order to analyze the CT number, expressed in HU, Romexis 4.5.1.R software (Planmeca, Finland) was used. For every patient, bone density was analyzed before and after photobiomodulation treatment, in the same areas of interest, within the same anatomical landmarks. Regarding metal artefacts, the analysis area was defined at an optimal minimum distance from the implant site, so as not to interfere with possible metal artefacts, which could generate abnormal bone density values.

The measurement’s focus on the trabecular bone, being the most homogeneous part of the maxillary bone and having the advantage of being described and characterized regarding HU values, with the help of conventional CT [32]. Moreover, the trabecular bone is the preferred area for placement of endosseous implants; therefore, changes at this level can influence the treatment plan, evolution, and prognosis of the case. We also performed measurements at the level of the vestibular cortical bone, where the area of interest did not receive any other type of treatment (for example, bone graft materials, which could act as a barrier for the laser radiation) than that of photobiomodulation.

**Figure 3:**
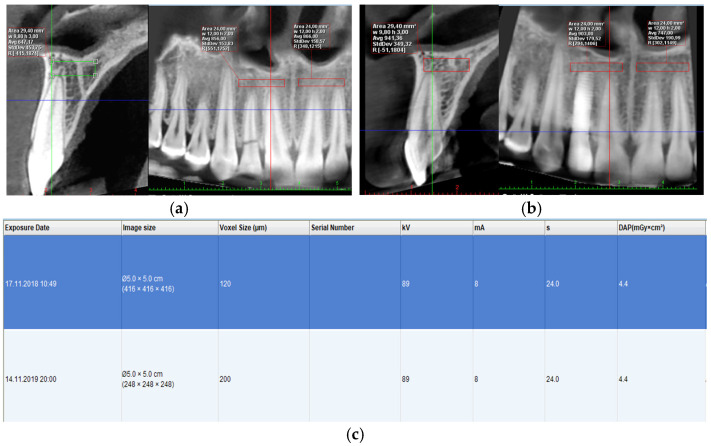
Initial CBCT view, definition of area of interest (**a**); post-surgical CBCT appearance after 12 months, same area of interest (**b**); parameters of acquisition of both CBCTs (**c**).

To localize the targeted sites and record the mean CT number, as well as the standard deviation, we used Romexis 4.5.1.R software (Planmeca, Finland). In each site, the region of interest is a rectangular bone volume, with an average area of 38.43 mm^2^ for the cortical area and an average area of 19.23 mm^2^ for the trabecular volume, corresponding to the alveolar ridge. A total of 18 cortical sites and 79 trabecular bone sites were analyzed. Every voxel in this volume is characterized by a CT number, expressed in HU. The software displays the mean value of CT numbers, their standard deviation, as well as range of HU values (lowest and highest HU values encountered in the analyzed volume) (Figure 3).

All the measurements made were analyzed statistically, the results being presented in the dedicated section. It was also made a comparison between the results obtained for the anterior maxilla and the posterior maxilla. The limit between the two areas is represented by the face distal of the maxillary canine on each side, respectively, the mesial face of the first premolar on each side.

## 3. Results

Patients included in the study group are presented in the table below, describing particularities related to sex, age, associated pathologies, and treated area (Table 1).

Each voxel in the beam computed tomography volume taper is characterized by a CT number, expressed in HU. Software displays the mean value of CT numbers, their standard deviation, as well as the range of HU values (lowest and highest HU values encountered in the analyzed volume).

**Figure 4:**
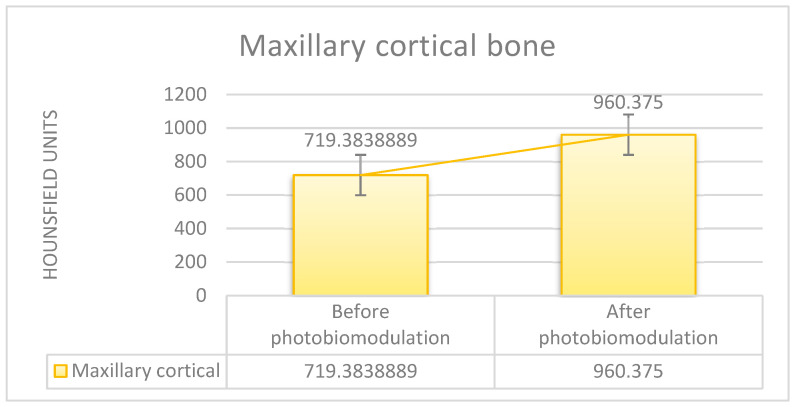
Average bone mineral density (study group), measured at the cortical level, in Hounsfield units, before of surgery and 1 year after surgery and laser PBM.

**Figure 5:**
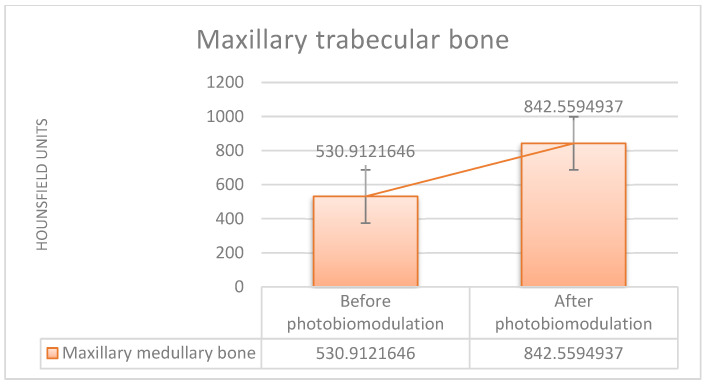
Mean bone mineral density (study group), measured in the trabecular bone, in Hounsfield units, before surgery and 1 year after surgery and treatment laser PBM.

The comparative evolution of bone mineral density in the study group, quantified by HU, at the level of the maxillary cortical bone, as well as in the trabecular bone, before and after treatment, for all patients included in the study, is represented in Figure 4 and Figure 5.

Based on the data analysis, the comparison between the laser and non-laser groups reveals that patients who underwent laser PBM showed a statistically significant improvement in bone mineral density, with the mean increasing from 530.91 HU before treatment to 842.55 HU after treatment (*t*-test: *p* < 0.001).

In contrast, the non-laser group showed no significant improvement, with a slight decrease in bone mineral density, as the mean dropped from 495.19 HU before treatment to 462.16 HU after treatment (*t*-test: *p* = 0.47). Table 2 summarizes the mean HU density results for the study group and the control group in the trabecular bone area, for which statistically significant differences were obtained. The table also shows the total number of measurements performed for the indicated areas.

**Figure 6:**
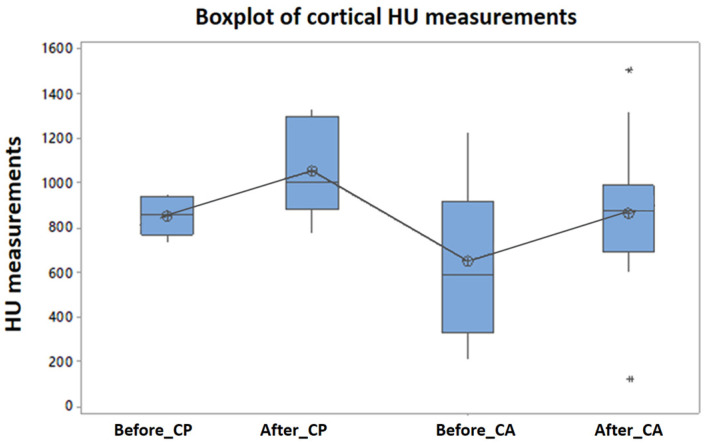
Tukey’s test applied to the HU indices measured at the cortical level, before and after the treatment of photobiomodulation, *p* = 3.6% (CA: cortical anterior, CP: cortical posterior) (study group). * denotes outliers.

In the study group, for both the female and male sex, the differences between the Hounsfield units measured at the level of the cortical bone, before and after treatment, comparatively, show significant statistical differences (ANOVA test Table 3 and Table 4, Tukey’s test Figure 6).

**Figure 7:**
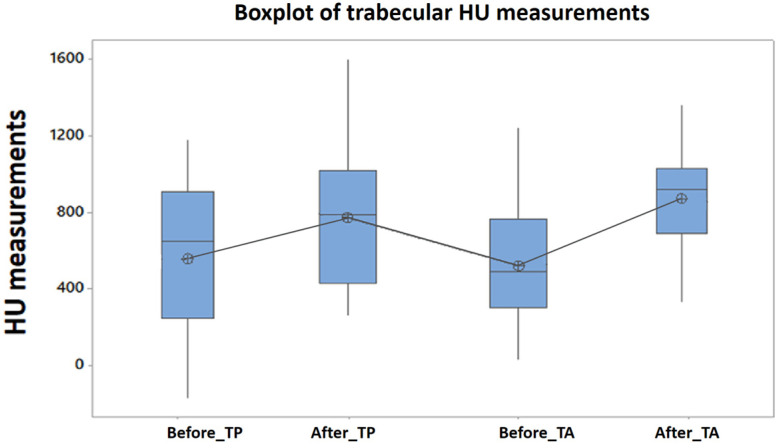
Tukey’s test applied to the HU indices measured in the trabecular bone, before and after the treatment of PBM, *p* = 0% (MA: medullar anterior, MP: medullar posterior) (study group).

**Figure 8:**
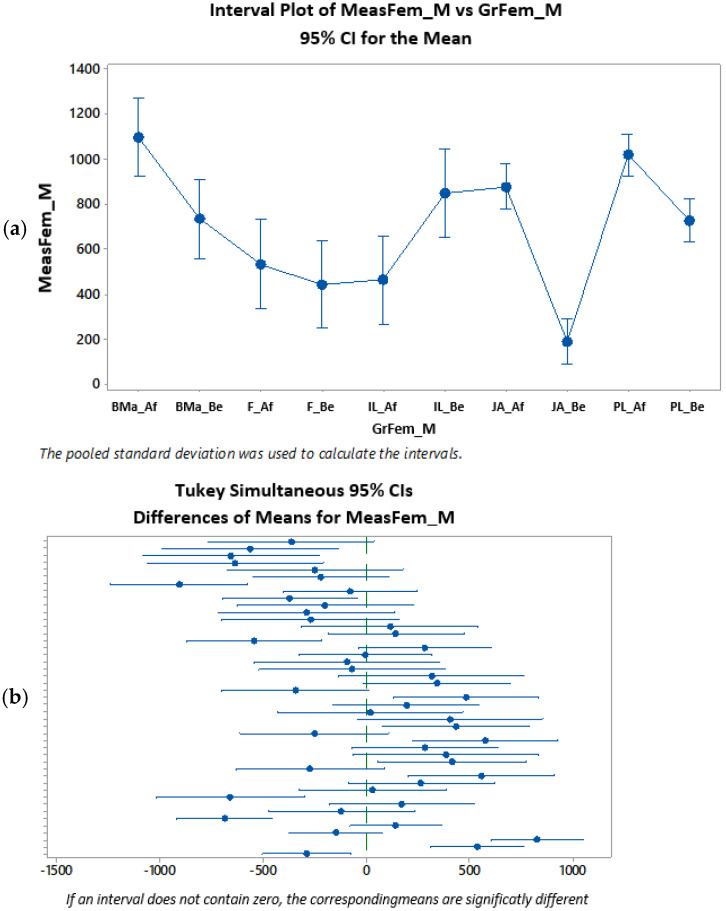

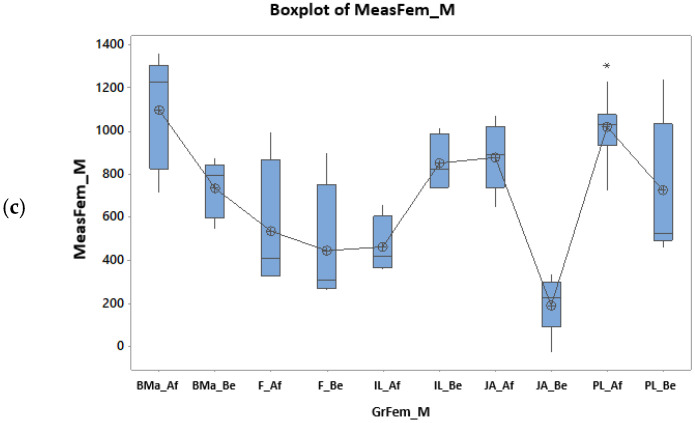
Statistical analysis of HU indices measured in the trabecular bone, before and after the treatment of PBM, for the women’s group (**a**–**c**) (Af = after, Be = before). * denotes outliers.

**Figure 9:**
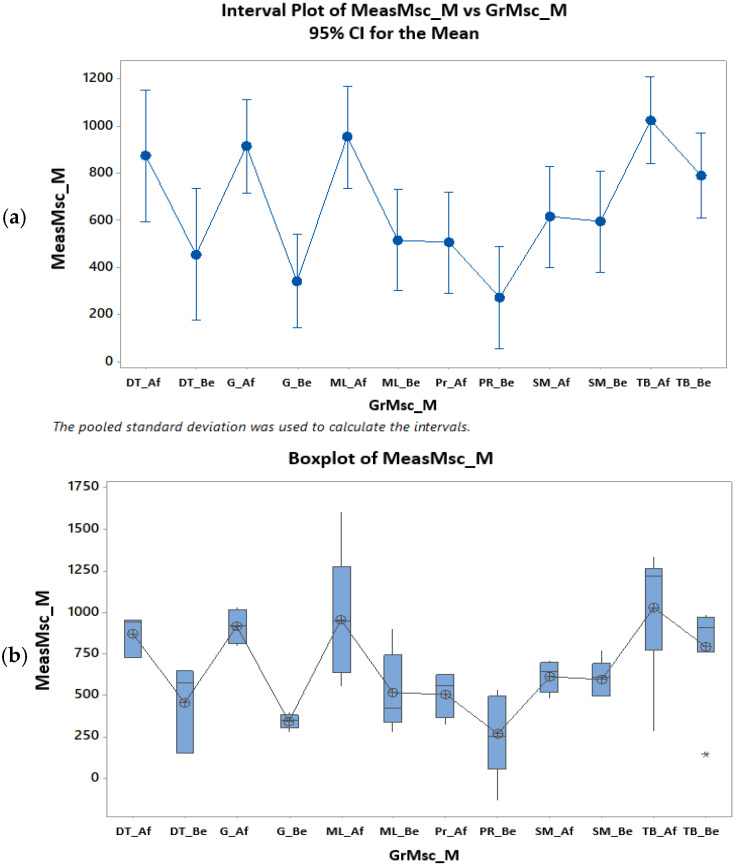
Statistical analysis of HU indices measured in the trabecular bone, before and after the treatment of PBM, for the men’s group (**a**) (if the intervals (vertical lines) overlap, there are no significant differences, if they do not overlap there are significant differences), (**b**) (patient G has very little dispersion before and quite little after; patient ML has quite large dispersion before and very large after) (Af: after, Be: before). * denotes outliers.

Similarly, the means of Hounsfield units measured at the level of the maxillary trabecular bone, before and after treatment, compared, show significant statistical differences, for both women and men (Anova test Table 5 and Table 6, Tukey test Figure 7, Figure 8 and Figure 9).

The comparison of anterior and posterior areas in terms of averages of Hounsfield units measured at the level of maxillary cortical bone, respectively, maxillary trabecular bone, does not show significant differences in terms of statistical analysis, neither for the study nor for the control group.

**Figure 10:**
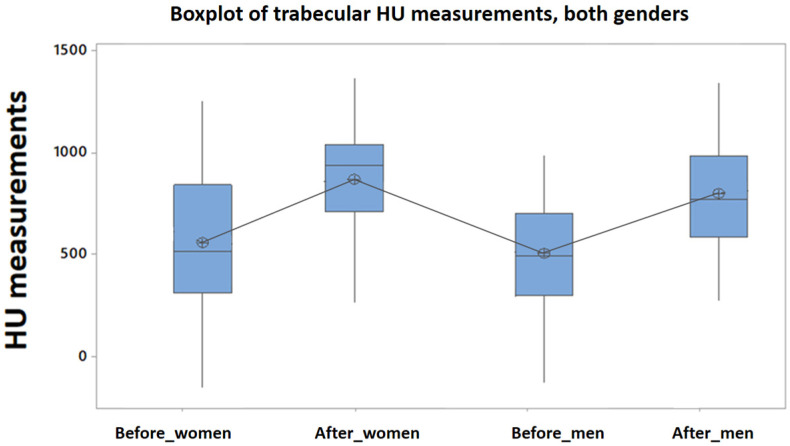
Tukey’s test applied to the HU indices measured in the trabecular bone, before and after the treatment of PBM, both in the case of the female and male patient groups (study group).

Regardless of the patient’s sex, both at the level of the cortical bone, as well as at the level of the maxillary trabecular bone, there are statistical differences significant before and after the treatment performed, in the study group (Figure 10).

### Power Analysis

Post hoc power analysis was conducted to assess the robustness of our results for the ANOVA tests, with males and females as the comparison groups. Using the following parameters: mean difference of 284.5, standard error of 70, and sample sizes n_1_ = 9 (male patients) and n_2_ = 18 (female patients), the calculated statistical power was 0.733, which is close to the desired threshold of 0.8. To achieve the optimal power of 0.8, a minimum of 11 male patients and 23 female patients would have been required.

## 4. Discussion

CBCT is used in the examination of the paranasal sinuses, in orthognathic surgery, in cases of trauma, in joint disease, to evaluate inflammation of the head and neck, to evaluate cysts and tumors, for endoscopic functional sinus surgery, and the complex examination of the sinuses [33,34]. CBCT is also used in surgery, endodontics and orthodontics, in the treatment planning of complex cases that require a multidisciplinary approach, in cases that require evaluation with 3D software, for measuring bone volume, area or morphometry, and bone density [35,36,37,38,39,40,41,42]. When CBCT has been used to assess bone density, the results found are different in many studies, one of the reasons being the variation in equipment and settings, such as the device type, image acquisition parameters, and the position of the evaluated tissue relative to the field of view [26,43,44,45]. By reconstructing the image with the help of the software, an HU value is associated to each voxel, known in the specialized literature as the CT number. It describes the ability of a substance to attenuate an X-ray beam, ranging from −1000 HU for air to about 3000 HU for enamel [46]. Each voxel is characterized by a CT number (voxel value), and software analysis of the CBCT image reports both the mean value and the standard deviation of the CT numbers belonging to the selected volume. The standard deviations of HU values obtained by CBCT can reveal differences between different types of trabecular bone, being a parameter that shows us the homogeneity of bone density at the level of the region of interest [45]. Despite the numerous variables that can affect image quality and the determination of gray values in CBCT examinations, great efforts have been made to describe methods capable of mathematically correcting these gray levels. For this purpose, X-ray attenuation coefficients from standardized materials were used as a reference [45,47,48,49] and even correction algorithms during or after image acquisition [50]. Even so, the comparisons between structures within the FOV have similar positions that seem viable; however, because the effects on the gray values in the regions of interest are the same [13], this is the reasoning taken into account in the present study. In order to objectively interpret the bone density, the fact that each axial CT image consists of 260,000 pixels, and each pixel has a CT Hounsfield number (Hounsfield HU unit), corresponding to tissue density, is taken into account. A high HU value indicates high bone density. Structures, such as calcification and bone, which absorb X-rays, are seen as being white and having a high HU value (80–100 HU). Scale range includes water reflected at a moderate level (0 HU), fat below zero (−80 HU), and air at the lowest values (−1000 HU) [51].

Numerous studies in the specialized literature have established the ranges of HU values corresponding to each bone density class, respecting the Misch classification [52]: D1: >1250 HU, D2: 850–1250 HU, D3: 350–850 HU, D4: 150–350 HU, and D5: <150 HU. A very interesting ex vivo study [53] aims to assess the reliability of NewTom 5G cone beam computed tomography (CBCT) in performing voxel gray value measurements using computed tomography (MSCT)-derived Hounsfield units (HUs) as the clinical reference (gold) standard. Gray values of CBCT voxels revealed a strong correlation with Hounsfield units obtained from MSCT. However, due to image artifacts resulting from scattering, gray values from CBCT scans demonstrated a higher mean value and larger standard deviations compared to Hounsfield values of MSCT. The same authors [54] investigated the ability of CBCT, compared to microCT, to microstructurally characterize bone tissue. Bone microarchitecture plays an important role not only in bone healing (the source of osteoblasts and osteoclasts) but also contributes to the strength of bone tissue. The most recommended microstructural parameters for estimating bone strength are trabecular thickness (Tb.Th), number (Tb.N), and the space between them (Tb.Sp). μCT is the gold standard modality for trabecular microstructural assessment. However, it can only evaluate small bone samples and cannot be used for clinical scanning of patients [18]. Thus, three qualitative parameters were followed in the cited study: trabecular number (Tb.N), thickness (Tb.Th), and separation (Tb.Sp). The results of the study demonstrated that the CBCT system (3D Accuitomo 170) used with the highest resolution available (80 μm), provides accurate measurements of bone microstructure, compared to μCT. The results suggest that CBCT can be an accurate tool for the analysis of bone microstructure before the insertion of a dental implant, taking into account that due to the voxel size, CBCT introduced overestimation in the measurement of Tb.Sp and Tb.Th.

Ogura et al. conducted a study [55] on 202 patients, using shades of gray in CBCT images to draw a correlation between the age of the patient and the bone density measured at the level of the mandibular cortex, in the region of the mental nerve foramen. The study concludes with the help of Pearson correlation test; this method is applicable for the evaluation of bone density, even if one cannot accurately correlate gray levels with Hounsfield units, as specified in other similar studies [22].

Another study carried out in the same direction [56] included 24 patients with type 2 diabetes, for which the bone density comparison was made measured on CBCT with that obtained by Dexa osteodensitometry (X-ray osteodensitometry-DEXA is considered the gold standard in the non-invasive assessment of bone density in orthopedics, endocrinology, and traumatology [57]), these being corroborated with the glycosylated hemoglobin level (HbA1c). This study demonstrated that CBCT is an effective tool in the diagnosis of osteoporosis, a positive correlation being established between DEXA T-score values and gray values measured on the CBCT images of edentulous mandibular arches, in the case of diabetic patients. These results are consistent with those of Barngkgei [58] and Güngör [59], who have compared DEXA and CBCT scans and concluded that osteoporosis can be predicted with high accuracy from the radiographic density value of the mandibular body.

Furthermore, Guerra et al. [60] systematically reviewed the literature regarding the ability of CBCT images to identify individuals with low bone density. They concluded that the density derived from CBCT–radiography is a promising tool for differentiating individuals with osteoporosis from individuals with a normal bone density. On the other hand, in an in vitro study, Hua et al. [22] have compared bone density values derived from DEXA analysis and density derived from CBCT gray values for the mandible and found no correlation. However, these selected studies used different CBCT devices and different voxel sizes, which drives us towards the already known hypothesis, according to which trabecular bone measurements and, consequently, image quality are affected by the parameter techniques, such as voxel size, the unit itself, voltage, and amperage tube and FOV selection [61]. In fact, as we mentioned before, these parameters represented inclusion/exclusion criteria in our study, finally making a comparison between identical FOVs, with same acquisition parameters.

A similar study [62], but targeting another pathology with bone tissue implications, used CBCT to determine bone density in patients with ectodermal dysplasia. They considered the evaluation scale in Hounsfield units. The study reports statistically significant differences between bone densities measured using Hounsfield units in patients with dysplasia ectodermal, compared to the control group, considering CBCT an appropriate tool for assessing bone density, in order to achieve an appropriate treatment plan.

In our study, the results obtained confirm the same hypothesis, proving that CBCT is a useful tool and suitable for measuring bone mineral density at the maxillary bone level. Similar results were obtained by David et al. [45], when analyzing voxel values of trabecular bone samples in CBCT, for implant planning purposes.

Moreover, in terms of the ability to detect statistically significant differences in patients who underwent laser PBM treatment after interventional oral surgery, laser therapy has been shown to increase bone density, both at the level of the vestibular cortical bone and at the level of the trabecular bone, independent of the patient’s sex and the anatomical region. This suggests that laser therapy played a pivotal role in enhancing bone density recovery; whereas, the absence of laser treatment did not yield meaningful improvements. The quantification of the PBM effect on bone mineral density using cone beam computed tomography allowed for the formulation of some clinical conclusions, in agreement with the experimental ones, obtained in the studies we have previously performed [9,10,63].

The number of clinical studies evaluating the effect of PBM laser therapy is limited, mainly due to the difficulty in evaluating the results. It is, therefore, all the more necessary to find valid clinical protocols that confirm the results previously obtained in the in vitro experimental studies. In a recent review, which was published in 2024 by Lu et al. [64], the authors were interested in the current clinical application of PBM devices in bone repair. From the 16 clinical studies included, from 2012 to 2023, 8 studies (the majority) had implemented the use of 806–830 nm diode lasers, followed by 4 studies using 660 nm diodes, 3 utilizing 980 nm, and 2 investigating 780 nm. Regarding the spot energy density measured in J/cm^2^, it varied among studies between 0.71 and 238.85, with the output power being situated between 40 and 300, the majority of them reporting acceleration of bone repair. Even so, the variability of the parameters is very high. As Zhu et al. [65] concluded earlier, in 2023, the irradiation dose is considered a significant light parameter affecting the effects of PBM. In 2018, Na et al. [66] showed in a very precise way the reaction of the osteoblast to different doses of light energy, as follows: for a 940 nm LEDs with an energy density of 1 J/cm^2^, osteoblast proliferation and osteoclast differentiation with bone repair activity take place in vitro, while a high dose over 5 J/cm^2^ will determine the opposite effects. A recent in vitro study performed by Vigliar et al. [67] in 2024 showed that laser PBM therapy contributed to improving the bone repair process in tibia defects filled with bovine biomaterial, when using an 830 nm laser with an energy of 6.2 J/cm^2^, similar to our previous animal studies. Also, in 2023, the group of researchers Rando et al. [68] published a review on the effects of PBM on alveolar bone repair. After analyzing the 18 studies meeting the inclusion criteria, several conclusions emerge, namely, lasers in the range 808–830 nm were of predominant use (7 studies of 18), and the energy ranged from 10 J/cm^2^ to 89 J/cm^2^, with 2 exceptions of 178 J/cm^2^ and 180 J/cm^2^. They also point out the difficulty in standardizing application protocols due to the diversity of equipment and scientific protocols among different research groups.

However, our study has few important limitations that must be considered. First, being a retrospective study, although the number of patients included may be acceptable for a preliminary study, it needs to be expanded to obtain a qualitative statistical analysis and a better sample size. Secondly, being a retrospective study also implies non-randomization, with possible baseline variations between patients. Thirdly, although we had very accurate inclusion criteria regarding general associated pathology, we should take into consideration a possible relation between bone mass quality and quantity in patients with cardiovascular disease. Last but not least, as we have shown in the previous paragraphs, the assessment of bone density using CBCT must be subject to rigor related to the acquisition parameters, so that the result obtained can be reliable.

## 5. Conclusions

According to the studies available to date, the indication of using CBCT as the examination of choice for the determination of bone mineral density, especially when the values obtained are compared with the preset standard values, was long discussed. The present study, based on a comparison over time, of the same patients, having the same acquisition parameters of CBCT images, demonstrated results with statistical significance regarding mineral bone density improvement of patients who underwent laser PBM treatment. This positive effect of laser therapy has been shown, both at the level of the vestibular cortical bone and at the level of the trabecular bone, independent of the patient’s sex and anatomical region. Quantification, using CBCT, of the effect of laser radiation on bone mineral density allowed for the formulation of some clinical conclusions, also highlighting the utility of cone beam computed tomography as a valuable predictive factor in assessing bone density. Thus, our study brings into discussion the clinical applicability of two aspects, as follows: firstly, the usefulness of laser PBM treatments for improving mineral density in the jaw bones, and secondly, the precautions that we must take into account when using CBCT as a tool for quantifying bone mineral density, especially regarding acquisition parameters. However, we consider this a pilot study, and we intend, based on the results obtained, to develop a comprehensive randomized clinical trial.

## Figures and Tables

**Table 1:** Study group description.

Nr. Crt.	Patient	Sex	Age	Systemic Disease/Medication	Implant Site
1.	T.B.	M	74	Ischemic heart disease	1.2, 1.4, 1.6, 2.2, 2.3, 2.4, 2.5
2.	D.B.	M	24	-	1.2
3.	B.D.	F	51	-	1.4
4.	B.M.	F	69	Arterial hypertension	1.2, 1.3, 2.2, 2.3
5.	B.M.	F	61	-	2.2, 2.3
6.	C.A.	F	63	Arterial hypertension	2.5, 2.6
7.	F.A.	F	72	-	1.4, 1.5
8.	G.I.	M	73	Heart failure	2.2, 2.3
9.	I.L.	F	60	Arterial hypertension	2.4, 2.6
10.	J.A.	F	60	-	1.5, 2.5, 2.6
11.	L.M.	M	40	Chronic hepatitis B	2.5
12.	P.I.	F	55	-	1.4, 1.6, 2.4, 2.5, 2.6
13.	P.R.	M	49	-	1.4
14.	S.M.	M	52	-	2.4

**Table 2:** Summary of average HU density results for the study and the control group.

	No. of Patients Included	No. of Measurements Performed	Pre-Operative Trabecular Mean HU Values	Post-Operative Trabecular Mean HU Values
Study group	14	79	530.91	842.55
Control group	14	112	495.19	462.16

**Table 3:** Anova test applied to the HU indices measured at the cortical level, before and after the treatment of photobiomodulation, for the women’s group (study group).

Group 1—F— cortical	Average difference	Standard error average	*p*
	378.4	114.4	0.011 < 0.05

**Table 4:** Anova test applied to the HU indices measured at the cortical level, before and after the treatment of photobiomodulation, for the group of men (study group).

Group 2—M— cortical	Average difference	Standard error average	*p*
	245.6	102.4	0.025 < 0.05

**Table 5:** Anova test applied to the HU indices measured in the trabecular bone, before and after the treatment of PBM, for the women’s group.

**Group 3—F—** **medullar**	Average difference	Standard error average	*p*
	254.1	75.8	1.28 × 10^−3^ < 0.05

**Table 6:** Anova test applied to the HU indices measured in the trabecular bone, before and after the treatment of PBM, for the group of men.

**Group 4—M—** **medullar**	Average difference	Standard error average	*p*
	284.5	70.0	1.56 × 10^−4^ < 0.05

## Data Availability

Data supporting reported results can be obtained from the corresponding authors.
